# Childhood Factors Associated With Unnatural Death Through Midadulthood

**DOI:** 10.1001/jamanetworkopen.2024.0327

**Published:** 2024-02-23

**Authors:** Kimberly B. Roth, Geoffrey Kahn, Carla L. Storr, Holly C. Wilcox

**Affiliations:** 1 Department of Community Medicine, Mercer University School of Medicine, Savannah, Georgia; 2 Center for Health Policy & Health Services Research, Henry Ford Health, Detroit, Michigan; 3 University of Maryland School of Nursing, Baltimore; 4 Department of Mental Health, Johns Hopkins Bloomberg School of Public Health, Baltimore, Maryland

## Abstract

**Importance:**

Life expectancy is decreasing in the US. Without national efforts to address factors that support policies and programs directed at children living in areas of concentrated poverty, life expectancy will likely continue to decline while costs and suffering associated with unnatural deaths will increase.

**Objective:**

To identify which childhood factors are associated with death from unnatural causes through midadulthood.

**Design, Setting, and Participants:**

For this cohort study, longitudinal data on childhood characteristics came from a group-randomized intervention trial implemented in Baltimore City Public Schools, Baltimore, Maryland (baseline 1985-1986; all students entering first grade were selected to participate at age 6 years). Participants were followed up to midadulthood with a National Death Index search through December 31, 2020. Data analysis was performed from February to May 2023.

**Exposures:**

Exposures included individual factors (ie, sociodemographic characteristics, teacher-reported aggressive behavior, self-reported depression, anxiety, early alcohol and cannabis use, and assaultive violence exposure), family and peer factors (ie, household structure and education level, deviant peer affiliation, and parental monitoring), and neighborhood factors (ie, rates of neighborhood assault and public assistance).

**Main Outcomes and Measures:**

The main outcome was unnatural death, defined as death due to unintentional injury, suicide, and homicide. A National Death Index search ascertained participants who died by age 41 to 42 years and cause of death. Multivariable Cox proportional hazards models were used to identify whether the exposures were independently associated with future mortality by unnatural causes.

**Results:**

The initial trial included 2311 children, and longitudinal data were available for 2180 participants (median [IQR] age in first grade, 6.3 [6.0-6.5] years; 1090 female [50.0%]; 1461 Black [67.0%]; 1168 received free or reduced lunch in first grade [53.6%]). A total of 111 male participants (10.2%) and 29 female participants (2.7%) died; among those who died, 96 male participants (86.5%) and 14 female participants (48.3%) died of unnatural causes. Two factors remained significantly associated with mortality from unnatural causes: female sex was associated with reduced risk (hazard ratio, 0.13; 95% CI, 0.08-0.22), and neighborhood public assistance was associated with increased risk (hazard ratio, 1.89; 95% CI, 1.09-3.30).

**Conclusions and Relevance:**

In this urban population-based cohort study, no modifiable risk factors of mortality at the level of the individual (eg, depression or anxiety and substance use) or the family (eg, household education level) were identified. However, the degree of neighborhood poverty in early childhood was significantly associated with death by unnatural causes in early adulthood, suggesting that economic policies are needed to advance health equity in relation to premature mortality.

## Key Points

**Question:**

Which childhood factors are associated with death from unnatural causes (unintentional injury, suicide, and homicide) by midadulthood in an urban population-based cohort?

**Findings:**

In this cohort study of 2180 participants, 10.2% of male participants had died by age 41 years. Only male sex and neighborhood poverty at ages 10 to 11 years remained significantly associated with unnatural death after controlling for other individual, family, and neighborhood factors.

**Meaning:**

These findings suggest that to make long-term gains in reducing health disparities, efforts targeting concentrated neighborhood-level poverty in childhood should be a national priority.

## Teaser

This cohort study examines childhood factors that are associated with death from unnatural causes through midadulthood.

## Introduction

In 2021, life expectancy decreased in the US, especially among Black and Indigenous individuals.^1^ Drug overdoses, homicide, suicide, and motor vehicle fatalities accounted for 31% of the decline in life expectancy from 2014 to 2021.^2,3^ Unintentional injury, suicide, and homicide, collectively termed *unnatural deaths*, remained the top 3 leading causes of death among those aged 15 to 34 years from 2001 to 2021, despite current preventive strategies.^4^ These deaths result in substantial psychological costs to bereaved family members.^5,6,7^ In 2019, the total costs arising from unnatural deaths in the US were $4.2 trillion.^4,8^ From a Life Course-Social Field perspective,^9^ incorporating factors from childhood from a variety of domains (eg, family, peers, and neighborhood), is vital for elucidating early targets for prevention.

The extant literature on factors associated with unnatural death is characterized by methodological limitations, such as a focus on high-risk populations,^10,11^ which limits generalizability, and cross-sectional and retrospective studies, which are prone to recall bias.^12^ Although childhood is an important developmental period for lifelong health,^13^ much etiological and intervention research is concerned with downstream risk factors more proximal to death. Few population-based prospective studies have examined childhood risk factors for unnatural death among urban samples of predominantly Black individuals, who are overrepresented in unnatural causes of death such as homicide.^14^ The suicide rate among Black individuals aged 10 to 24 years increased by 37% from 2018 to 2021, greater than any other racial or ethnic group.^15^ Research often focuses on specific types of unnatural deaths, despite evidence of shared risk factors across homicide, suicide, and overdose.^16^

Few cohort studies have reported childhood factors associated with unnatural death, and existing studies report mostly on risk factors at the individual or family level.^16,17,18,19,20,21,22^ Friedman et al^20^ followed the prospective cohort of Terman et al^23^ of primarily White, gifted children growing up in California and found that childhood conscientiousness and social dependability were associated with longevity. Loeber et al^12^ studied factors associated with homicide death in the Pittsburgh Youth Study and found that a combination of early behavior problems, large family size, and family relationship problems was associated with homicide. A prospective study of a birth cohort in Finland found that young male participants with divorced parents were more likely to die by unintentional injury or suicide after controlling for psychiatric diagnosis and parental social class.^24^ However, the number of deaths in these studies was quite small.

Given the growing evidence that health outcomes are largely influenced by social determinants of health,^25^ a major limitation of the current body of literature is the lack of focus on neighborhood and contextual influences^26^ and the lack of inclusion of Black individuals. In this study, we longitudinally follow a cohort of children in a city with a history of racial segregation and lack of investment in Black communities that has impacted the concentration of poverty and opportunities of Black residents.^27,28,29,30^ The aim of this study was to identify modifiable childhood risk factors for unnatural death using a social-ecological and life course framework^31^ by including factors from several levels—individual, microsystem (family and peers), and mesosystem (neighborhood)—across several life stages (middle and late childhood and adolescence).

## Methods

Data for this cohort study are from a group-randomized prevention trial^32,33^ in 19 Baltimore City Public schools located in 5 distinct urban areas in East Baltimore, Maryland, that varied in sociodemographic factors and degree of racial segregation. All students in 2 cohorts of children entering first grade during the 1985 to 1986 (1196 students) and 1986 to 1987 (1115 students) academic years were randomized to 2 years of exposure to the Good Behavior Game, Mastery Learning intervention or the standard classroom setting (control condition). Childhood and adolescent assessments were conducted in grades 1 through 9. Baseline or childhood assessments were missing for 130 youths, and 1 child was excluded because of missing date of birth information (eFigure in Supplement 1). In young adulthood, 75% of the sample participated in a follow-up interview (ages 20-21 years). Death data were obtained from the National Death Index (NDI) Plus^34^ through December 31, 2020, which provided linkage of study participants data to vital statistics data from the 50 states. The following identifiers were linked to the NDI: last name, first name, sex, date of birth, and Social Security number. The NDI provided multiple potential matches of deaths to each submitted person, and a scale ranking the quality of information was created, allowing us to limit matches according to quality.

Study protocols were approved by the institutional review board of Johns Hopkins University. Parents provided written informed consent for data collection. Verbal assent was obtained from youths; after they reached adulthood, they provided written consent. This study followed Strengthening the Reporting of Observational Studies in Epidemiology (STROBE) reporting guideline.^35^

### Individual Factors

#### Early Childhood

Sociodemographics (collected from school files in fall of first grade) included sex, race (Black or all other races), and free or reduced lunch status. Data on race were included to describe the demographics of the population; because Black individuals constituted the majority of the sample, we used this as one group and combined the rest of the sample. Teacher-rated aggressive behavior was assessed in the fall of first grade using the Teacher Observation of Classroom Adaptation–Revised.^36^ A trained interviewer administered this structured interview to the teacher, who was able to rate each child’s performance in several domains. Ten items, rated on a scale from 1 (almost never) to 6 (almost always), comprised the aggressive-disruptive subconstruct: breaks rules, breaks things, harms others, harms property, fights, yells at others, lies, acts stubborn, teases others, and takes others’ property. Children scoring in the top quartile were considered highly aggressive and disruptive.

Depression was assessed via child self-report with the Children’s Depression Inventory, and anxiety was assessed with the Revised Children’s Manifest Anxiety Scale in fall of first grade.^37,38,39^ The Children’s Depression Inventory contained 27 items on a scale of 0 to 2 assessing frequency of depressive symptoms in the past 2 weeks. The Revised Children’s Manifest Anxiety Scale contained 37 yes-or-no items on the child’s anxious feelings or actions. Items were summed for each scale, and children scoring above the median on both depressive and anxiety symptoms were classified as high depressed or anxious, consistent with prior research.^40^

#### Later Childhood and Adolescence

Reported drug use was assessed from ages 9 to 14 years. Youths were asked whether they had ever used alcohol, cannabis, crack, cocaine, or sniffed any substance and, if so, age of first use. Because of the low prevalence of use of crack, cocaine, and inhalants, a 3-category variable was created to indicate early substance use (before age 15 years): no use, either alcohol or cannabis, or both alcohol and cannabis.

Lifetime exposure to assaultive violence (rape; sexual assault; shot or stabbed; mugged or threatened with a weapon; held captive, tortured, or kidnapped; and badly beaten) was retrospectively assessed in early adulthood (ages 20-23 years). Participants were asked to report the age of occurrence and frequency of each type of traumatic event.

### Household and Peer (Microsystem) Factors

#### Early Childhood

Parents reported on household structure and highest level of education in the household in an interview in third grade. Household structure was characterized as having 1 adult in the home vs 2 or more. Education level was dichotomized into non–high school graduate vs high school graduate or higher.

#### Later Childhood and Adolescence

In fifth grade, deviant peer affiliation (defined as formation of attachments to deviant and substance-using peers)^41^ was measured through 5 standardized interview questions adapted from the Peer Behavior Scale,^42^ and parental monitoring (defined as parental tracking of the child’s whereabouts, activities, and adaptations)^43^ was measured with 10 standardized questions adapted from the Parental Monitoring Scale.^42^ Children indicated the frequency with which their peers engaged in deviant behavior, such as substance use and antisocial activities, and the level of parent or guardian supervision. Total scores were created for both measures by summing responses. Higher scores indicate more deviant peer affiliation and more parental monitoring, respectively.

### Neighborhood (Mesosystem) Factors in Later Childhood and Adolescence

Neighborhood rates of aggravated assault and public assistance were measured by 1990 US Census data and were used to describe neighborhood context when participants were aged approximately 10 to 11 years (grade 5 or 6). Raw data were transformed by taking the arcsine square root, owing to the small proportions after the data were weighted by the number of people within a Census tract, and then multiplying by 10. Intervention design status (control vs intervention) and cohort (1 or 2) were also included.

### Outcomes

Cause of death was from the NDI and, when not available, death certificates were obtained. Deaths were classified according to manner of death categories—natural, unintentional injury, suicide, homicide, or undetermined—according to what was written on the death certificate and/or *International Classification of Diseases, Ninth Revision* or *International Statistical Classification of Diseases and Related Health Problems, Tenth Revision (ICD-10)* codes (eTable in Supplement 1). In this study, all undetermined deaths were classified as unnatural owing to notes on the death certificate indicating narcotic intoxication without clear evidence of suicide intent (15 deaths); however, 2 undetermined deaths, described as “subject ran over by train” and “gunshot wound to the head,” were reclassified as suicides.

### Statistical Analysis

Data analysis was performed from February to May 2023. Missing data on covariates ranged from 3% to 47% (median, 23%) and were multiply imputed simultaneously in R statistical software version 3.4.3 (R Project for Statistical Computing) for all covariates in the final analytic sample using the multiple imputation by chained equations method^44,45^ and package.^46^ Each covariate with missing values was modeled as a function of all other variables, plus the event indicator and estimated cumulative hazard, as recommended in White et al.^47^ The number of iterations was set to 10, and the mean and SD plots were examined to ensure imputations converged. Ten imputed data sets were generated.

All covariates of interest were first analyzed independently. Kaplan-Meier curves were examined, log-rank tests computed, proportional hazard assumption evaluated, residual plots examined, and univariate Cox proportional hazards models were run to estimate the relative hazard of death. For all covariates, models were run with an interaction with time to assess whether the interaction should be included to account for nonproportional hazards. Children entered the analysis at approximately age 6 years in the fall of first grade. Robust SEs were clustered at the school-grade classroom level. Deceased participants exited the analysis at their date of death, with those dying of natural or unknown (missing) causes censored. In addition, 4 individuals with deaths before age 10 years were censored at date of death, all of which were due to unintentional injury. Alive participants were censored at either the age of last interview or the date through which NDI deaths were ascertained (December 31, 2020). The median (IQR) age at the time of NDI death ascertainment was 41.0 (40.5-41.6) years.

Multivariable models were then run in the following order: including all individual-level factors as covariates, then adding in family and peer factors, and finally adding neighborhood-level factors, which resulted in the full, final model. All analyses were conducted using Stata statistical software version 14 (StataCorp)^48^ using both the *mi* and *st* suite of commands for multiply imputed survival data. Statistical significance was assessed at the *P* < .05 level, and all hypothesis tests were 2 sided.

## Results

A total of 2180 participants (1090 female [50.0%]; 1461 Black [67.0%]; 1168 receiving free or reduced lunch in the fall of first grade [53.6%]) (data not shown) were included in the analysis. The median (IQR) age in first grade was 6.3 (6.0-6.5) years. In total, 140 of 2180 participants (6.4%) died by December 31, 2020 (Table 1), 110 (78.6%) due to unnatural causes, including 64 homicides (45.7%), 25 unintentional injuries (17.9%), 15 drug-related of undetermined intent (10.7%), and 6 suicides (4.3%). Two deaths were by unknown cause. The median (IQR) age of death was 27.7 (21.9-34.0) years and varied by cause of death (Table 2). Natural (median [IQR] age at death, 32.0 [26.4-35.4] years) and opioid-related (median [IQR] age at death, 31.7 [22.7-38.4] years) deaths occurred up to 10 years later than unintentional injuries (median [IQR] age at death, 23.5 [16.6-30.3] years), suicides (median [IQR] age at death, 24.1 [18.6-33.6] years), and homicides (median [IQR] age at death, 26.7 [22.2-31.9] years). Sex, free or reduced lunch status, and ever experiencing assaultive violence were significantly associated with cause of death. Male participants were significantly more likely than female participants to die (111 male participants [10.2%] vs 29 female participants [2.7%]; χ^2^_1_ = 51.3; *P* < .001) especially from unnatural causes (96 male participants [86.5%] vs 14 female participants [48.3%]; χ^2^_1_ = 16.1; *P* < .001). The most common causes of death were homicide (61 participants [55.0%]) among male participants and natural causes among female participants (13 participants [44.8%]). Unintentional injuries were the most common cause of unnatural death among female participants (7 participants [24.1%]) (Table 2). Black participants were much more likely to die from homicide than participants of any other race (60 participants [93.8%] vs 4 participants [6.3%]), whereas participants of any race other than Black were more likely than Black participants to die from opioid overdose (11 participants [73.3%] vs 4 participants [26.7%]). Individuals who qualified for free or reduced lunch were more likely to die by suicide or homicide, and less likely to die by unintentional injury or opioid overdose. Those exposed to violence exclusively died by homicide or opioids. Among family and peer factors, household structure was the only variable significantly associated with cause of death, with those in single-adult households more likely to die from homicide compared with those in households with 2 or more adults. Finally, both neighborhood characteristics (aggravated assaults and public assistance) were significantly associated with overall and specific-cause mortality.

**Table 1.** Vital Status and Cause of Death Categories in the Sample Overall and by Sex

Characteristic	Participants, No. (%)		
	Overall (N = 2180)	Male (n = 1090)	Female (n = 1090)
Vital status^a^			
Alive	2040 (93.6)	979 (89.8)	1061 (97.3)
Deceased	140 (6.4)	111 (10.2)	29 (2.7)
Age at death, median (range) [IQR], y	27.7 (7.0-41.6) [21.9-34.0]	27.1 (7.0-41.6) [22.1-33.6]	31.6 (7.4-40.8) [21.7-35.5]
Cause of death^b^^,^^c^			
Natural	28 (20.0)	15 (13.5)	13 (44.8)
Unnatural	110 (78.6)	96 (86.5)	14 (48.3)
Unintentional injury	25 (17.9)	18 (16.2)	7 (24.1)
Suicide	6 (4.3)	5 (4.5)	1 (3.5)
Homicide	64 (45.7)	61 (55.0)	3 (10.3)
Opioid	15 (10.7)	12 (10.8)	3 (10.3)
Unknown	2 (1.4)	0	2 (6.9)

^a^ χ^2^_1 _= 51.3 (*P* < .001).

^b^ Percentages are calculated using deceased participants as the denominator.

^c^ χ^2^_1 _= 16.1 (*P* < .001).

**Table 2.** Age at Death and Childhood Characteristics of Analytic Sample by Vital Status and Cause of Death^a^

Characteristic	Vital status			Cause of death^b^					
	Alive, No. (%) (n = 2040)	Deceased, No. (%) (n = 140)	*P* value	Natural, No. (%) (n = 28)	Unintentional injury, No. (%) (n = 25)	Suicide, No. (%) (n = 6)	Homicide, No. (%) (n = 64)	Opioid, No. (%) (n = 15)	*P* value
Age at death, median (range) [IQR], y	NA	27.7 (7.0-41.6) [21.9-34.0]	NA	32.0 (17.0-40.8) [26.4-35.4]	23.5 (7.0-39.8) [16.6-30.3]	24.1 (17.6-39.0) [18.6-33.6]	26.7 (15.5-41.6) [22.2-31.9]	31.7 (17.7-40.3) [22.7-38.4]	NA
Individual factors									
Age at grade 1 entry, mean (SD), y	6.28 (0.45)	6.32 (0.45)	.29^c^	6.38 (0.42)	6.34 (0.60)	6.26 (0.57)	6.33 (0.41)	6.18 (0.39)	.71
Sex									
Male	979 (48.0)	111 (79.3)	<.001^d^	15 (53.6)	18 (72.0)	5 (83.3)	61 (95.3)	12 (80.0)	<.001
Female	1061 (52.0)	29 (20.7)		13 (46.4)	7 (28.0)	1 (16.7)	3 (4.7)	3 (20.0)	
Race									
Any race other than Black	683 (33.5)	36 (25.7)	.06	6 (21.4)	11 (44.0)	4 (66.7)	4 (6.3)	11 (73.3)	<.001
Black	1357 (66.5)	104 (74.3)		22 (78.6)	14 (56.0)	2 (33.3)	60 (93.8)	4 (26.7)	
Lunch status									
Paid	963 (47.2)	49 (35.0)	.005	11 (39.3)	16 (64.0)	2 (33.3)	11 (17.2)	9 (60.0)	<.001
Free or reduced	1077 (52.8)	91 (65.0)		17 (60.7)	9 (36.0)	4 (66.7)	53 (82.8)	6 (40.0)	
Aggressive behavior									
Low	1474 (76.8)	85 (63.4)	<.001	18 (64.3)	18 (75.0)	4 (66.7)	33 (55.9)	10 (66.7)	.58
High	446 (23.2)	49 (36.6)		10 (35.7)	6 (25.0)	2 (33.3)	26 (44.1)	5 (33.3)	
Depression or anxiety									
Low	1257 (75.3)	83 (74.1)	.77	17 (81.0)	14 (66.7)	4 (80.0)	41 (80.4)	6 (50.0)	.20
High	412 (24.7)	29 (25.9)		4 (19.0)	7 (33.3)	1 (20.0)	10 (19.6)	6 (50.0)	
Early substance use									
No early use	782 (49.9)	57 (49.6)	.93	11 (50.0)	14 (60.9)	4 (80.0)	23 (43.4)	3 (30.0)	.31
Either alcohol or marijuana	690 (44.0)	50 (43.5)		8 (36.4)	7 (30.4)	1 (20.0)	27 (50.9)	7 (70.0)	
Both alcohol and marijuana	95 (6.1)	8 (7.0)		3 (13.6)	2 (8.7)	0	3 (5.7)	0	
Assaultive violence									
No	968 (78.1)	46 (71.9)	.25	13 (100.0)	7 (100.0)	1 (100.0)	20 (58.8)	4 (50.0)	.01
Yes	272 (21.9)	18 (28.1)		0	0	0	14 (41.2)	4 (50.0)	
Family and peer (microsystem) factors									
Household structure									
≥2 Adults in household	788 (63.1)	35 (55.6)	.23	11 (78.6)	6 (66.7)	0	12 (38.7)	6 (75.0)	.04
Single adult	461 (36.9)	28 (44.4)		3 (21.4)	3 (33.3)	0	19 (61.3)	2 (25.0)	
Highest household education									
Not high school graduate	429 (32.5)	27 (39.7)	.21	5 (35.7)	4 (44.4)	0	15 (41.7)	3 (37.5)	.97
High school graduate or higher	893 (67.5)	41 (60.3)		9 (64.3)	5 (55.5)	0	21 (58.3)	5 (62.5)	
Deviant peers score at grade 5, mean (SD)	10.01 (3.80)	10.70 (4.08)	.09	10.83 (4.15)	10.45 (4.03)	9.00	11.56 (4.15)	7.91 (2.50)	.09
Parental monitoring score at grade 5, mean (SD)	33.39 (5.06)	32.54 (5.29)	.15	31.07 (6.98)	34.60 (4.45)	41.00	32.14 (4.50)	33.20 (6.05)	.23
Neighborhood (mesosystem) factors									
Aggravated assault rate, mean (SD)^e^	0.95 (0.40)	1.12 (0.42)	<.001	1.12 (0.44)	1.05 (0.41)	0.80 (0.41)	1.26 (0.36)	0.69 (0.35)	<.001
Public assistance rate, mean (SD)^e^	2.57 (1.24)	3.10 (1.25)	<.001	3.08 (1.36)	2.70 (1.26)	2.28 (1.35)	3.57 (1.01)	1.95 (1.23)	<.001
Intervention group									
Control	1178 (57.8)	79 (56.4)	.89	15 (53.6)	12 (48.0)	4 (66.7)	35 (54.7)	11 (73.3)	.57
Good Behavior Game	403 (19.8)	30 (21.4)		5 (17.8)	5 (20.0)	1 (16.7)	18 (28.1)	1 (6.7)	
Mastery Learning	459 (22.5)	31 (22.2)		8 (28.6)	8 (32.0)	1 (16.7)	11 (17.2)	3 (20.0)	
Cohort									
1 (1985-86)	1085 (53.2)	72 (51.4)	.69	15 (53.6)	15 (60.0)	5 (83.3)	31 (48.4)	5 (33.3)	.25
2 (1986-87)	955 (46.8)	68 (48.6)		13 (46.4)	10 (40.0)	1 (16.7)	33 (51.6)	10 (66.7)	

Abbreviation: NA, not applicable.

^a^ Percentages for categorical variables with missing values do not include missing values.

^b^ Excludes 2 participants with unknown cause of death.

^c^ *P* values for continuous variables are from *t* tests or analysis of variance.

^d^ *P* values for categorical variables are from χ^2^ tests.

^e^ Neighborhood rates of aggravated assault and public assistance were measured by 1990 US Census data and were used to describe neighborhood context when participants were aged approximately 10 to 11 years (grades 5 or 6). Raw data were transformed by taking the arcsine square root, owing to the small proportions after the data were weighted by the number of people within a Census tract, and then multiplied multiplying by 10.

Table 3 displays results from the Cox survival analysis models. Among the univariate models, free or reduced lunch status, high aggression, single-adult family structure, and neighborhood aggravated assault and public assistance rates were associated with significantly increased hazards of unnatural death. Black race, experiencing assaultive violence, and deviant peer affiliation did not significantly increase the hazard of unnatural death. Being female was associated with a significantly reduced risk of death from unnatural causes, but having a high school or higher household education was not. However, after adjusting for individual-level factors, being female was the only significant protective factor. High aggression and household structure were no longer associated with hazard of death. Adding family and peer factors to the model eliminated free or reduced lunch status as a significant factor. In the final model, being female remained the only factor significantly associated with a reduced risk of mortality (hazard ratio, 0.13; 95% CI, 0.08-0.22), and neighborhood-level public assistance was the only factor significantly associated with increased risk of mortality (hazard ratio, 1.89; 95% CI, 1.09-3.30).

**Table 3.** Relative Hazard of Unnatural Death From Univariate and Multivariable Cox Regression Models

Variable	Univariate model		Adjusting for individual factors^a^		Adjusting for individual plus family and peer factors^b^		Adjusting for all factors^c^	
	HR (95% CI)	*P* value	HR (95% CI)	*P* value	HR (95% CI)	*P* value	HR (95% CI)	*P* value
Individual factors								
Female sex (reference, male)	0.13 (0.08-0.23)	<.001	0.14 (0.08-0.23)	<.001	0.13 (0.08-0.23)	<.001	0.13 (0.08-0.22)	<.001
Black race (reference, any race other than Black)	1.39 (0.95-2.02)	.09	1.18 (0.76-1.83)	.45	1.20 (0.76-1.89)	.43	0.66 (0.36-1.20)	.17
Free or reduced lunch (reference, paid)	1.79 (1.25-2.58)	.002	1.64 (1.09-2.45)	.02	1.44 (0.93-2.24)	.11	1.03 (0.64-1.6,)	.90
High aggression (reference, low aggression)	1.91 (1.26-2.88)	.002	1.33 (0.87-2.03)	.19	1.31 (0.85-2.01)	.22	1.38 (0.92-2.07)	.12
High depression or anxiety (reference, low depression or anxiety)	1.12 (0.70-1.80)	.63	1.14 (0.72-1.81)	.58	1.10 (0.69-1.75)	.69	1.13 (0.71-1.79)	.61
Either alcohol or cannabis (reference, no early use)	1.11 (0.71-1.73)	.65	1.00 (0.65-1.53)	.98	1.01 (0.64-1.60)	.97	1.03 (0.65-1.64)	.90
Both alcohol and cannabis (reference, no early use)	0.99 (0.39-2.52)	.99	0.65 (0.25-1.74)	.39	0.64 (0.23-1.76)	.38	0.61 (0.22-1.73)	.35
Ever assaultive violence (reference, never)	1.87 (1.00-3.51)	.05	1.78 (0.94-3.35)	.08	1.72 (0.91-3.27)	.09	1.74 (0.91-3.33)	.09
Family and peer (microsystem) factors								
Single adult in household (reference, ≥2 adults)	1.79 (1.11-2.86)	.02	NA	NA	1.39 (0.81-2.39)	.23	1.22 (0.71-2.10)	.47
High school graduate or higher (reference, not high school graduate)	0.61 (0.37-1.00)	.05	NA	NA	0.76 (0.43-1.32)	.32	0.83 (0.47-1.46)	.51
Deviant peers	1.05 (1.00-1.11)	.07	NA	NA	1.01 (0.95-1.07)	.84	1.00 (0.94-1.07)	.98
Parental monitoring	0.98 (0.93-1.03)	.38	NA	NA	1.02 (0.97-1.08)	.47	1.03 (0.97-1.08)	.36
Neighborhood (mesosystem) factors								
Neighborhood aggravated assault rate	2.46 (1.59-3.79)	<.001	NA	NA	NA	NA	0.47 (0.11-2.02)	.31
Neighborhood public assistance rate	1.39 (1.21-1.60)	<.001	NA	NA	NA	NA	1.89 (1.09-3.30)	.02
Good Behavior Game or Mastery Learning intervention (reference, control)	1.04 (0.73-1.48)	.82	NA	NA	NA	NA	1.04 (0.77-1.41)	.79
Cohort 2 (reference, cohort 1)	1.22 (0.86-1.73)	.27	NA	NA	NA	NA	1.26 (0.92-1.74)	.15

Abbreviations: HR, hazard ratio; NA, not applicable.

^a^ *F*_81,123.0_ = 7.78 (*P* < .001).

^b^ *F*_12,972.1_ = 5.43 (*P* < .001).

^c^ *F*_16,1789.8_ = 6.55 (*P* < .001).

## Discussion

In this cohort study, 10.2% of male participants had died by age 41 years. Similar numbers of male participants and female participants died of natural causes, but male participants were much more likely to die by unnatural causes, especially homicide. Several individual, family, and neighborhood factors were associated with unnatural death, but only male sex and Census tract–level concentration of poverty remained significantly associated with unnatural death after controlling for other factors. Our finding that female sex is protective against unnatural death is consistent with national mortality data. In the US, male individuals accounted for 67% of the 224 935 preventable injury-related deaths in 2021.^49^ However, mechanisms underlying the gender gap in mortality are not fully understood. Several biological and social mechanisms, as well as modifiable behaviors, such as the use of alcohol and drugs and violence, may account for much of the shorter life expectancy among men.^50^ Our findings that sociodemographic and community variables, rather than modifiable individual-level factors (eg, behavior), were independently associated with unnatural death is consistent with a prior study,^12^ but longitudinal research identifying childhood factors associated with death is rare.

The current study offers several advantages. First, its longitudinal design allows for investigation of temporality, because we followed 2 cohorts of individuals for approximately 35 years (from ages 6 to 41 years). This is particularly important when looking at outcomes such as suicide or homicide, where recall bias can severely impact inferences through the use of proxy-based recall after death.^12^ Second, the sample was submitted to the NDI, with *ICD-10* cause of death information obtained. Third, the sample originated from an urban area and included predominantly Black individuals, a population at increased risk of unnatural death.^14^

Our results offer modest support for less traditional prevention approaches. As was pointed out by Rockett and colleagues,^51^ current prevention initiatives in the US emphasize downstream approaches, such as identifying and treating people in crisis; however, without parallel national efforts to address upstream factors and inequity, including policies and programs directed at children living in a high concentration of poverty (eg, increasing the federal minimum wage and earned income tax credits),^52^ life expectancy will likely continue to decline while costs and suffering associated with unnatural deaths will increase. Given the enormous costs and suffering associated with unnatural deaths, states should enact policy to address childhood poverty, as has been done in Maryland.^53,54^ In addition to strengthening economic supports, promoting connectedness and teaching coping and problem-solving skills have shown crosscutting benefits across violence and injury domains.^55,56^ In 2021, suicides and homicides reached record highs, and from 2019 to 2021, nearly all of the increases in the US homicide and suicide rates were explained by an increase in the use of firearms.^57^ Gun-related homicide and suicide rates increased by 44% from 2014 to 2021^3^; in 2021, more than 47 000 US individuals died by firearms.^58^ States should implement evidence-based policies shown to reduce gun-related suicides, accidents, and homicides,^57^ such as permit to purchase laws,^59^ extreme risk protection orders,^60^ and child access prevention laws.^61,62^

### Limitations and Strengths

There are several limitations to our results. Examination of factors by specific cause of death was hampered by rare occurrence of specific causes of death (eg, suicides). In addition, assessing suicide by death certificate often underrepresents the true suicide prevalence because suicidal intent is difficult to establish.^63,64,65^ Death ascertainment via the NDI is not perfect, and matches can range in certainty.^34^ Because the parent study was not designed to focus on mortality, some important constructs were not assessed. Our findings may not be generalizable to all life stages, settings, or populations. In addition, factors were measured at a single time point, but most can change over time (eg, families move and family structure and neighborhood characteristics can change). Furthermore, our understanding of the meaning of constructs may also change over time (eg, a teacher’s report of student aggression can be influenced by cultural bias).

Despite these limitations, our study capitalized on prospective cohort data, which is important when studying unnatural death. As was pointed out by Loeber et al,^12^ most homicide studies are based on information collected after a homicide. Longitudinal studies are important to provide information years or, in our case, decades before death, past the peak period of homicide risk in late adolescence to early adulthood.^12^ Our study has multi-informant prospective data starting in childhood at a median age of 6 years to elucidate the impact of early life experiences on later unnatural death. Objective death ascertainment was not subject to loss to follow-up. Furthermore, ascertainment occurred into the participants’ 40s, therefore exceeding the highest risk period of most causes of unnatural death. In addition, our sample included a majority of Black individuals, which is a strength.

## Conclusions

Since the early 2000s, there has been a marked increase in research on social determinants of health to elucidate the role of factors beyond individual-level variables. In this cohort study, neighborhood poverty at ages 10 to 11 years was the only significant, independent, modifiable factor associated with unnatural death. To make long-term gains in reducing health disparities, efforts targeting concentrated neighborhood-level poverty in childhood should be a priority.

## Notes

Supplement 1. **eFigure.** Study Flow Chart **eTable.** *ICD* Recodes and *ICD* Codes for Categories of Natural and Unnatural Death

Supplement 2.
Data Sharing Statement
