# Supplementary material for: Childhood Factors Associated With Unnatural Death Through Midadulthood
Source: JAMA Netw Open. 2024 Feb 23;7(2):e240327. doi: 10.1001/jamanetworkopen.2024.0327 (PMC10891468; doi:10.1001/jamanetworkopen.2024.0327)
Supplement: Supplement 1. — eFigure. Study Flow Chart eTable. ICD Recodes and ICD Codes for Categories of Natural and Unnatural Death [file jamanetwopen-e240327-s001.pdf]

Supplemental Online Content

Roth KB, Kahn G, Storr CL, Wilcox HC. Childhood factors associated with unnatural death through midadulthood. *JAMA Netw Open*. 2024;7(2):e240327. doi:10.1001/jamanetworkopen.2024.0327

eFigure. Study Flow Chart

eTable. *ICD* Recodes and *ICD* Codes for Categories of Natural and Unnatural Death

This supplemental material has been provided by the authors to give readers additional information about their work.

© 2024 Roth KB et al. *JAMA Network Open.*

eFigure. Study Flow Chart

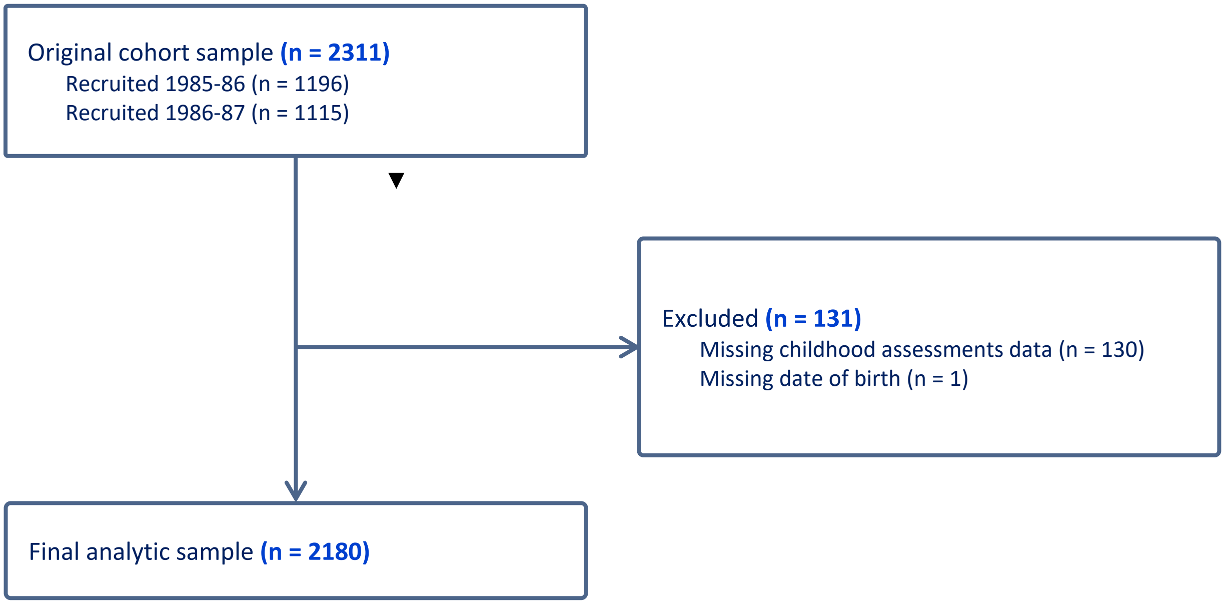

© 2024 Roth KB et al. *JAMA Network Open.*

eTable. *ICD* Recodes and *ICD* Codes for Categories of Natural and Unnatural Death

Category	ICD-9 Recodes*	ICD-9 Codes	ICD-10 Recodes**	ICD-10 Codes
**Natural**	00100 to 29800	001-799	001 to 111	A01-R99
**Unnatural**	-	-	-	-
Unintentional Injury	30000 to 33600	E800-E949	112 to 123	V01-X59,Y85-Y86
Suicide	33700 to 34400	E950-E959	124 to 126	*U03,X60-X84,Y87.0
Homicide	34600 to 34900	E960-E969	127 to 129	*U01-*U02,X85-Y09,Y87.1
Undetermined Intent	35200 to 35700	E980-E989	131 to 133	Y10-Y34,Y87.2,Y89.9

*Refers to the 282 ICD-9 recodes of selected causes of death.
**Refers to the 113 ICD-10 recodes of selected causes of death.

© 2024 Roth KB et al. *JAMA Network Open.*
