# Supplementary material for: Childhood Factors Associated With Unnatural Death Through Midadulthood
Source: JAMA Netw Open. 2024 Feb 23;7(2):e240327. doi: 10.1001/jamanetworkopen.2024.0327 (PMC10891468; doi:10.1001/jamanetworkopen.2024.0327)
Supplement: Supplement 2. — Data Sharing Statement [file jamanetwopen-e240327-s002.pdf]

Data Sharing Statement

Roth. Childhood Factors Associated With Unnatural Death Through Midadulthood. *JAMA Netw Open*. Published February 23, 2024. doi:10.1001/jamanetworkopen.2024.0327

Data
Data available: Yes
Data types: Deidentified participant data
How to access data: Email to obtain permission from corresponding author
When available: With publication

Supporting Documents
Document types: None

Additional Information
Who can access the data: researchers whose proposed use of the data has been approved
Types of analyses: approved projects
Mechanisms of data availability: after approval of a proposal and with a signed data access agreement
Any additional restrictions: none
